# New Methods to Address Old Challenges: The Use of Administrative Data for Longitudinal Replication Studies of Child Maltreatment

**DOI:** 10.3390/ijerph14091066

**Published:** 2017-09-15

**Authors:** Emily Hurren, Anna Stewart, Susan Dennison

**Affiliations:** School of Criminology and Criminal Justice, Griffith Criminology Institute, Griffith University, Mt Gravatt, 4122, Australia; a.stewart@griffith.edu.au (A.S.); susan.dennison@griffith.edu.au (S.D.)

**Keywords:** child maltreatment, administrative data, longitudinal, replication

## Abstract

Administrative data are crucial to the “big data” revolution of social science and have played an important role in the development of child maltreatment research. These data are also of value to administrators, policy makers, and clinicians. The focus of this paper is the use of administrative data to produce and replicate longitudinal studies of child maltreatment. Child protection administrative data have several advantages. They are often population-based, and allow longitudinal examination of child maltreatment and complex multi-level analyses. They also allow comparison across subgroups and minority groups, remove burden from individuals to disclose traumatic experiences, and can be less biased than retrospective recall. Finally, they can be linked to data from other agencies to explore comorbidity and outcomes, and are comparatively cost and time effective. The benefits and challenges associated with the use of administrative data for longitudinal child maltreatment research become magnified when these data are used to produce replications. Techniques to address challenges and support future replication efforts include developing a biographical understanding of the systems from which the data are drawn, using multiple data sources to contextualize the data and research results, recognizing and adopting various approaches to replication, and documenting all data coding and manipulation processes. These techniques are illustrated in this paper via a case study of previous replication work.

## 1. Introduction

Administrative data, data collected during the day-to-day operations of an organisation, are crucial to the “big data” revolution of social science [1]. The “…inevitable increase in the use of administrative data in child maltreatment research” was accurately predicted almost two decades ago [2] (p. 308). Since that time, the availability of digital administrative data from statutory child protection agencies has buoyed the literature base, particularly as these data enable researchers to revisit and address old research challenges. 

The focus of this paper is on the capacity of administrative data to mitigate the challenge of both producing and, perhaps more importantly, replicating longitudinal studies of child maltreatment. To begin, we provide the theoretical and empirical justification for longitudinal studies of child maltreatment and discuss the strengths and challenges associated with the use of administrative data for these studies. We then extend this discussion to focus on the need for longitudinal replications, and the use of administrative data for these replications. We conclude by highlighting a range of techniques that can be used to address the identified research challenges and make suggestions for future research. To illustrate key arguments, we present our own replication work as a case study.

## 2. Longitudinal Research: Needs and Past Challenges

Over recent decades there has been increasing recognition that children’s experiences of maltreatment must be understood longitudinally and temporally [3]. Longitudinal and temporal depictions of child maltreatment allow for consideration of the impact of maltreatment dimensions such as timing, frequency, and type, as well as their overlaps. This is important because different types of maltreatment (physical, sexual, and emotional abuse and neglect) may differentially increase the risk of various developmental outcomes [4]. Likewise, maltreatment experiences may have a different impact on developmental outcomes depending upon their timing across the life-course [5]. Reported figures indicate that a considerable percentage of maltreated children experience more than one type of maltreatment over their life-course [6,7]. Some studies indicate that the greater the number of maltreatment types or events experienced across the life-course, the greater the risk of negative developmental outcomes [8]. Clearly, longitudinal studies of child maltreatment are crucial to understanding the experience of maltreatment across the life-course, and associated developmental outcomes. Unfortunately, knowledge of their importance does not necessarily make longitudinal studies easier to produce. 

There are a variety of challenges associated with the production of longitudinal studies. The first hurdle is access to suitable data. Connelly, Playford, Gayle, and Dibben [1] distinguish between “made data” and “found data”. Traditional social science data sources fall within the category of “made data”. These are data that are collected or produced for the purpose of research. Made data often result from questionnaires or observational studies. Comparatively, “found data” are not collected for research purposes, but can, nonetheless, be utilised for research. Administrative data are an important component of “found data”, and can also be classified as “big data” [1].

Prior to the digitisation and application of administrative data, longitudinal studies of child maltreatment typically relied upon “made data”, and represented a considerable investment of time and resources. Data accounting for a person’s life-course experiences of maltreatment either took a lifetime to collect, or were retrospective in nature. Retrospective studies typically relied on participant recall, which could have a negative impact on the accuracy of the data. In addition, these studies were financially and ethically challenging. Under these conditions, it is not surprising that many researchers were either unwilling or unable to conduct longitudinal studies of maltreatment and its outcomes. 

Fortunately, the digitisation of administrative data and the increasing quality of the data collected have produced a time- and cost-effective means for conducting longitudinal research on child maltreatment. Further, the quality and quantity of administrative data continues to improve as computer technology continues to improve [2], and some jurisdictions are improving their data access processes [1]. 

In the following sections, we describe administrative child protection data and discuss the advantages and challenges associated with the use of administrative data for longitudinal studies of child maltreatment. We then extend our discussion to focus on the use of administrative data in longitudinal replication studies.

## 3. What are Administrative Data?

As noted briefly above, administrative data are data that are collected across the daily functioning of an agency. Though administrative datasets from statutory child protection agencies may differ across distinct jurisdictions as a function of legislative, policy, or procedural variations, there are some details that could be considered “typical”. Child protection administrative datasets typically include details regarding any maltreated child or young person having contact with the agency, such as their date of birth, gender, and ethnicity, and details regarding the maltreatment itself, such as type(s) and date(s) of notification. Records also typically include details of any investigation and its outcomes, such as substantiation decisions and interventions by the department or agency. Finally, some datasets also include details of the parent, guardian, or person responsible for the maltreatment. As records are typically kept for individuals over time, and stored under their name or a unique numeric identifier code, these data are able to be aggregated and viewed longitudinally, meaning, all system contacts of an individual across their life-course. 

## 4. Administrative Data for Longitudinal Child Maltreatment Research: Advantages and Challenges 

### 4.1. Advantages

There are eight generally acknowledged advantages associated with the use of administrative data for longitudinal studies of child maltreatment. First, these datasets typically contain the whole population of individuals known to a system or agency [1,2,9]. It is accepted that actual rates of maltreatment are underestimated within administrative data due to underreporting or challenges with investigation and substantiation [2,9,10]. Nonetheless, administrative data do include all known cases of substantiated maltreatment within a jurisdiction, and therefore represent the individuals who can be targeted by interventions. Additionally, when coupled with population figures such as the number of births in a jurisdiction during a particular year, or the number of children and young people in a particular population, administrative data can be used to develop statistical approximations of the prevalence of substantiated maltreatment within a particular population (for example, see [11]). This is especially helpful in jurisdictions that do not have formal incidence studies. 

Second, as administrative data are large and population based, they allow examination of comparison groups and minority groups [1,2]. For example, researchers can compare subgroups in relation to maltreatment type or timing, or victim gender. In addition, administrative data allow for the examination of vulnerable groups or groups normally excluded or lost from follow-up in standard longitudinal studies [1]. The examination of minority groups is an especially important area of focus in current child maltreatment research due to their overrepresentation in many statutory child protection systems (for example, see [11]). Large sample sizes also allow for the examination of gender-race intersectionality [12]. Recent intersectionality research suggests that to understand child maltreatment pathways, gender and race should be examined simultaneously, with analyses illustrating variations across distinct gender-race subgroups (for example, see [12,13]). 

Third, administrative data are “big data” [1]. They are multi-level, longitudinal, and historical [2]. These data enable a much larger range of complex analytical techniques than allowed by smaller samples [2]. Likewise, the capacity to test for complex interaction effects increases alongside the size of the data. In this sense, administrative data enable researchers to develop and test increasingly complex conceptualisations of maltreatment, and its precursors and outcomes, which can in turn better inform policy and practice. Most jurisdictions now have administrative data that span multiple birth cohorts. These data allow pre- and post-test analyses [2], examination of change over time (including the impact of policy change), and can allow researchers to distinguish between age and cohort effects [1]. Administrative child protection data also often contain more than one level of data [2]. Specifically, these data can include details of the victim and the perpetrator/person responsible. This can facilitate inclusion of multiple variables in analyses that might be excluded from standard data collections, and examination of the intergenerational nature of child maltreatment [2,9]. The intergenerational nature of child maltreatment is an important point of focus in current research [14,15]. In sum, these data can facilitate new contributions to this field of research [9]. 

Fourth, administrative data from multiple distinct agencies can be linked at the individual level using a variety of data linkage techniques [2,9]. Data linkage across distinct agencies allows researchers to examine the developmental sequelae or outcomes of child maltreatment, and explore how child maltreatment fits within the life-course of individuals who may have multiple and complex risks and needs (for example, see [11]). Examples could include justice, mental health, or education agencies. 

Fifth, administrative data are unobtrusive and may be less biased [9]. Specifically, these data remove the need for disclosure (particularly of traumatic events) by victims or perpetrators, which has a clear ethical advantage [1]. Additionally, administrative data typically stem from an investigation or assessment by a departmental worker. Hence, these data may be less affected by stigma, recall bias, or socially desirable reporting by victims or perpetrators [9], which are common problems in longitudinal research relying solely on retrospective recall. 

Sixth, administrative data are cost effective [1,2], particularly as they are “found” and not “made”. However, researchers have noted that there can be long delays in accessing these data, and they do require data management [1,10]. This can result in financial costs for some researchers.

Seventh, much child maltreatment research has been affected by inconsistent definitions of maltreatment and its subtypes, both in terms of definitions assigned by researchers and interpretations of respondents [3]. As administrative data are typically collected and recorded to be consistent with legislative or policy definitions, these large bodies of data are likely consistent in relation to the categorisation of particular experiences as distinct maltreatment types. By extension, when definitions change in response to policy or legislative changes, it is typically possible to identify the exact point at which definitions or categorisation changed, and the reasons behind these changes. In turn, researchers can monitor the impact of these changing definitions on research findings.

Eighth, and finally, administrative data enable the divides between research, policy, and practice to be bridged [2]. These data can benefit researchers, administrators, policy makers, and clinicians, and by extension vulnerable children and their families. They include cases that meet the criteria of abuse or neglect or harm of that particular jurisdiction [10], and relate specifically to the functioning of an agency. These data also enable evaluation [9,10], plus development or change of policy and interventions [1,10]. Hence, these data can be used to produce a range of policy-relevant research that has benefits for researchers, practitioners, and policy makers alike. 

For example, to date, administrative data have been used to develop tools for structured decision making and risk assessment [16]. Administrative data have also been crucial to identifying overrepresentation of racial minority groups [17] and children with disabilities [18] in child protection systems, and guiding appropriate responses to this overrepresentation. These data can also be used within departments for performance measurement [19], to assess service needs and requirements across regions and to determine appropriate staffing and resource requirements. Importantly, administrative data can be used to guide the provision of services to children, families, and jurisdictions most in need. Hence, these data can benefit researchers, policy makers, and clinicians, with the broader outcome being improved outcomes for vulnerable children and families.

### 4.2. Challenges

As with all research methodologies and data sources, the use of administrative data for longitudinal research can be challenging and is associated with some limitations. There are six commonly reported challenges. First, as noted by numerous researchers, child protection administrative data typically provide an underestimation of actual rates or experiences of child maltreatment [2,9,10]. Specifically, administrative data typically include only those cases in which maltreatment was recognised and reported. However, it can also be argued that policy and interventions can only be directed towards “known” cases of maltreatment, meaning these data are particularly suited to policy relevant research. 

Second, researchers have no control over the variables included in or excluded from the dataset, or control over how the data are collected, entered, and stored [2,9]. There can be data entry errors, and data quality can vary across jurisdictions [10]. Important variables can also be absent. For example, administrative child protection data often lack information on the socio-economic status of victims and perpetrators [9], meaning these variables cannot be included in many administrative studies, despite being theoretically important. Additionally, the recording of multiple maltreatment types can vary across jurisdictions. Some jurisdictions will record all maltreatment types present at the time of notification or assessment (multi-type maltreatment), while others will only report a primary or most severe maltreatment type [10]. Not all jurisdictions will include data on perpetrators, and some jurisdictions will include the “person responsible” for the child, though this person may not necessarily be the perpetrator [10]. These variations impact the breadth of studies that rely on administrative data, and can also create challenges in producing comparisons or replications across different jurisdictions. 

Third, it can be very difficult to monitor the migration or attrition of individuals within administrative datasets, which is important for longitudinal research [10,11]. Some families affected by maltreatment may be highly mobile, moving from one jurisdiction to another. It may be difficult to distinguish between cases in which maltreatment has ceased, and cases in which a family has moved beyond the jurisdiction of focus. 

Fourth, administrative data require considerable data management [1,2,10]. These data are rarely “analysis ready” upon receipt [10]. These data are not collected for research purposes [1,9], so researchers must often restructure the dataset [1,10], as well as recode and create variables [1]. In some instances, a single agency may store their data on multiple data systems or across multiple data sets, requiring linkage of records within a single agency at the individual level [1]. These processes can be challenging, complex, and time-consuming [1]. 

Fifth, researchers must invest time and effort into developing an understanding of the data systems and datasets before they can meaningfully analyse the data. Research using administrative data requires a thorough understanding of the system or agency from which the data are obtained, what population is represented, and what key variables represent [1,2,10]. When provided with administrative data from an agency or department, it is important to also request documentation in relation to the methods used to extract the data, as well as metadata files. In addition, it is important to liaise with key stakeholders who have knowledge of the data systems and datasets [1]. These details can prove useful in contextualising the data and performing quality assurance. 

Sixth, and finally, there are legal and ethical challenges associated with the use of administrative data, particularly in relation to the fact that they are used without informed consent from the individuals within the dataset [1,9]. In some cases, there can be long delays before data are obtained [1,10]. Additionally, after the data are obtained, researchers will not typically have “ownership” of the data [1,2]. Regardless, it is essential that researchers can maintain confidentiality, privacy, and data security [9].

### 4.3. Summary

There is a clear need for longitudinal research in the child maltreatment literature base. We, alongside other researchers, argue that the benefits of administrative data outweigh the challenges [9,10]. Researchers need not be dissuaded by the challenges associated with the use of administrative data. Interestingly, as noted by Brownell and Jutte, based on their particular benefits, some researchers have “…argued that there is a moral and economic obligation to make use of population-based administrative data…” [9] (p. 122). 

The evolution of child maltreatment research studies is testament to the developing awareness and growing sophistication in conceptualisation and understanding of consequences of maltreatment. With large data repositories and increasingly sophisticated analytic techniques, researchers are able to perform complex and multilevel analyses. Hence, researchers can explore concepts such as cumulative and interactive risk, as well as contextual factors stemming from various levels of the developmental system. In addition, heterogeneity can be better explored, and separate models be produced to separately examine gender-race subgroups. This moves the field beyond the acceptance that gender and race are important variables, to a focus on how these variables affect individuals, and to determine whether different interventions are required for different subgroups. 

Of course, these data are not simply of benefit to researchers, but also to administrators, policy makers, and clinicians. In the words of Jonson-Reid and Drake [20] (p. 392) “…child welfare policy can now be informed by a much more complete understanding of who we serve, how they are served over time, what other social service systems they encounter, and what outcomes they commonly experience”. It is hoped that the future will bring increased data quality and collaboration between researchers, administrators, policy makers, and clinicians to ensure improved child protection responses and, most importantly, better outcomes for vulnerable children and families.

Though researchers have shown a growing acceptance of the value of administrative data for child maltreatment research, very little discussion has been devoted to the value of administrative data for longitudinal replication studies. With newer and more complex techniques now available, we argue that conclusions from the past several decades of child maltreatment research should now be revisited in order to test the generalisability of these conclusions over time, and to extend our understanding wherever possible. The need for longitudinal replication studies in child maltreatment, and the use of administrative data for these longitudinal replications is the focus for the remainder of this paper. 

## 5. Longitudinal Replication Studies: Needs and Past Challenges

Social scientists have long acknowledged the importance of replication studies. In particular, replication studies provide a valuable test of the generalisability of research findings and allow greater confidence in research conclusions and recommendations. As noted by McNeeley and Warner [21], the results of a study may be affected by the particular methods used and the context of the study.

There are many approaches to replication in social science. For example, McNeeley and Warner [21] described two categories of replication: direct replication, in which the same methods, measures, and populations are used, and empirical generalisation, in which the same methods and measures are applied to a different population. According to this classification, direct replications test internal validity, while empirical generalisations test generalisability [21]. Crandall and Sherman [22] alternatively distinguished between exact (or direct) replication and conceptual replication. They argued that exact replications apply methodologies that are as close to the original study as possible, while conceptual replications apply alternative methodologies but test the same theoretical process. 

Replications of longitudinal studies in the child maltreatment literature are particularly important because child maltreatment policies and practices vary across place and time. It is generally accepted that child protection legislation and tertiary responses vary across different jurisdictions, or within some jurisdictions over time. As noted by Connelly, Playford, Gayle, and Dibben [1], policy contexts can be ever changing. For example, the considerable growth in Australian child protection systems has been partially attributed to a variety of factors such as the introduction of mandatory reporting, an increasingly risk-averse culture, increasing system capacity, broadening conceptualisations of maltreatment and harm, and a growing expectation that the government will play a role in the protection of children [23]. Additionally, child protection systems exist within and are affected by broader social systems that are themselves diverse and fluid. For example, using a variety of data sources, Finkelhor and Jones [24] found declining rates of child maltreatment and victimisation (including physical abuse, sexual abuse, and neglect) across the period 1993 to 2004. Importantly, though they noted the potential impact of system changes such as changes to notifications and investigations, they also noted the potential contribution of factors associated with broader social-level change. They particularly emphasised the likely impact of pharmacological treatments, social interventions, and economic prosperity [24]. 

There are also sound theoretical frameworks that highlight the value of replication for longitudinal child maltreatment research. For example, developmental systems theories [25], and the developmental and life-course criminology theoretical framework [26] each acknowledge the likelihood of variation across individuals, groups, time, and place, and highlight the need to understand the context in which life experiences occur. These theoretical perspectives clearly acknowledge the need for replication and the use of multiple sources of data [25,26]. 

Though their importance is generally accepted, published replication studies are relatively rare in the social sciences [21]; this is particularly true of longitudinal replication studies. The rarity of published replication studies could be attributed to reduced willingness of researchers to conduct these studies in the first instance and/or a reduced rate of acceptance of replication studies for publication [21]. Regardless, it is imperative to replicate research that informs policies and practices pertaining to vulnerable and at-risk groups, particularly when the consequences of ineffective policies may include the loss of life and the waste of scarce resources [21]. As child maltreatment research has considerable policy implications, and the result of inappropriate interventions include serious long-term consequences or even death, we argue that replication studies in this field are crucial. 

## 6. Administrative Data for Longitudinal Replications: Advantages and Challenges

The above-described advantages and challenges associated with the use of administrative data for longitudinal studies of child maltreatment continue to apply when using these data for replication studies. Importantly though, these advantages and challenges are multiplied in the process of replication.

### 6.1. Advantages

As argued by Drake and Jonson-Reid, “one critical advantage of administrative data that is not yet fully realized is the ability to replicate and alter the parameters of prior work” [2] (p. 310). As noted in the preceding section, there are many ways of performing a replication. Specifically, replication can incorporate applying the same analytical methods on the same populations, or the same methods on different populations, or can use different methods whilst assessing the same theoretical pathways.

Exciting replication possibilities facilitated by administrative data include the ability to perform theoretical replications across distinct jurisdictions, and methodological replications within distinct jurisdictions. For example, because administrative child protection data can typically be extracted based on date of birth of individuals, or dates of notifications and substantiations, these data can be used to compare distinct birth cohorts in a single jurisdiction over time, or a consistent birth cohort across multiple jurisdictions. These data can also be used to test the impact of interventions/policy changes by assessing variations in notification and substantiation trends pre- and post-policy change. Additionally, as administrative data can be linked to data from other agencies, they can also be used to assess variations in outcomes following intervention or policy change, or across cohorts. At the conclusion of this paper we present a case study of a replication to explore child maltreatment and youth offending links within and across birth cohorts. 

The replication opportunities offered by administrative data are numerous. However, these replications are not easy to do. Nor is it a simple task to draw reliable, reasonable, and policy-relevant conclusions. We discuss these challenges in more detail next. 

### 6.2. Challenges and Suggestions for Future Research

Though variations across time and place provide the rationale for longitudinal replication, they simultaneously make longitudinal replication studies using administrative data more challenging. For example, if researchers wish to perform a replication within a single jurisdiction across time (for example, a cross-cohort comparison), they must understand and account for the changing policy context over time, the broader social change of the jurisdiction, and how this may impact on the system and changes to departmental processes including computer systems and legacy databases. 

If researchers wish to perform a replication across distinct jurisdictions, they must account for variations in legislation and policy that may affect data entry and coding for each jurisdiction under examination. They must also account for contextual factors that may impact maltreatment rates and outcomes differently across each of these jurisdictions. Further, though many jurisdictions now have life-course or longitudinal data for multiple birth cohorts, due to the relatively late availability of computerised records in some jurisdictions [2], the cohorts available for scrutiny may vary from one jurisdiction to another. In short, the challenges associated with the use of administrative data for longitudinal studies of child maltreatment are magnified in replication attempts. 

Though there are challenges and limitations associated with the use of administrative data for longitudinal replication studies, the benefits are still overwhelming. Further, to date, there are no alternative data sources that can equal the breadth and complexity of analyses enabled by longitudinal administrative data. Fortunately, there are techniques that researchers can use to assist replication attempts. 

First, Connelly, Playford, Gayle, and Dibben [1] recommend having a biographical understanding of the system from which the data were drawn, as this will enable an understanding of the data as well as an understanding of change over time. Researchers can use multiple data sources to contextualise administrative data and any research results. For example, child protection agencies often report annual cross-sectional figures regarding notifications and substantiations within their jurisdiction. These figures can be compared over time to better understand changing patterns over time. Departmental annual reports may also include details of policy or system changes, and legislative changes also tend to be well documented. Together these data sources can be used to illustrate the context in which the administrative data were collected, as well as illustrate the way system changes may have impacted on the data and research results. 

Second, as noted earlier, there are many types of replication. Researchers can provide details of their method of replication, a rationale for their selection, and discuss the strengths and weaknesses of their approach. Third, to facilitate replication it is important to document all coding processes [10], and where possible share syntax and documentation [1]. These are important components of research transparency and integrity. Fourth, research conclusions and policy implications drawn from replication studies using administrative data are best presented in a manner that appropriately reflects the strengths and limitations of the data sources, analyses, and replication techniques. Connelly, Playford, Gayle, and Dibben [1] argue that the substantive importance of results should be considered alongside their statistical significance. Likewise, Hindman [27] suggests a range of techniques to ensure better statistical models and replication in social science using big data. We argue that these suggestions are relevant to research on child maltreatment that relies upon administrative data. To illustrate the above points, in the next section we present a case example of our own replication attempts.

## 7. Case Example: Replication Using Linked Administrative Data

Our research team has used linked administrative data from the Queensland child protection and youth justice systems for a considerable period of time [11]. One of our most recent studies was a longitudinal replication study using linked child protection and youth justice administrative data from Queensland [28]. The original study we sought to replicate was conducted by members of our team, and examined the links between child maltreatment and youth offending [29]. The original study used linked longitudinal population-based administrative data from the Queensland child protection (birth to 18 years) and youth justice (age 10 years to 17 years) systems for individuals born in 1983/1984. The data were analysed using the semi-parametric group-based method of trajectory analysis [29,30]. The analyses revealed six distinct trajectories of child maltreatment across the life-course (birth to 18 years), and differential proportions of youth offenders associated with each of these distinct trajectory groups. To test the generalisability of these results, we replicated the methodology using a newer birth cohort from Queensland [28]. Specifically, we linked comparable longitudinal, population-based administrative data from the Queensland child protection and youth justice systems for individuals born in 1990. We analysed the data using the semi-parametric group-based method of trajectory analysis, and attempted to compare the results across the 1983/1984 and 1990 cohorts. 

Our replication supported many of the results of the original study. For example, across both studies six distinct trajectories of child maltreatment were identified [28,29]. Additionally, maltreatment frequency often appeared to peak at ages that coincided with transition points, namely the transition to primary school and the transition to secondary school [28,29]. Finally, the trajectories in which maltreatment continued into or began during adolescence contained higher proportions of offenders, as did trajectories that extended across more than one developmental period [28,29]. Replication of key findings provides a stronger base for policy change. For example, the results of this replication provide a rationale for direction of services to at-risk adolescents, who may not have been targeted previously [31]. Our replication was also able to extend the results of the original study by highlighting the potential impact of multi-type maltreatment, overlaps across maltreatment dimensions, and their interactions with gender and race [28]. 

We encountered five key challenges in performing this longitudinal replication study, and used a range of techniques to address these challenges. First, the legislation guiding the functioning of the Queensland child protection system had changed in the year 1999. We examined each dataset to determine whether the change in legislation had impacted on the types of data available and the coding of key variables. Fortunately, our examination of the datasets indicated that the legislative change had resulted in minimal impact on the datasets that would affect our intended analyses. This process allowed us to have greater confidence in the replicability of the analyses.

Second, the data system used by the Queensland child protection system had changed while the 1990 cohort were still under the jurisdiction of the child protection department (i.e., before the age of 18 years). At the time that our data requests were being processed, data for our target cohort were being held across two different databases. Though access to data on out-of-home placements for the individuals in our dataset would have been preferable, access to these data would have required additional work-arounds and linkage by the department. As out-of-home placements were not crucial to the original study, we opted to forgo these data in the data extraction for the 1990 cohort. This meant we were unable to account for the potential impact of out-of-home care on the links between child maltreatment and youth offending. We acknowledged that this was a limitation in the study. Nonetheless, the absence of these variables had no impact on the replication process.

Third, for both cohorts we had data only on the most serious harm type present, or in other words, the maltreatment type most responsible for the harm or risk of harm. We did not have data on all maltreatment types present at the time of notification or substantiation, as these data were not included in the data extraction from the child protection department. During the approximately eight years between the publication of the original study and the performance of the replication study, the child maltreatment research base had shifted to include a greater focus on the experience of multi-type maltreatment. Though this was not an important point of focus in the original study, we were required to alter the focus of the replication study to ensure continued relevance to the current literature base and policy environment. This meant that within our replication study we created a variable that provided a conservative estimation of the experience of multi-type maltreatment (i.e., cases where the recorded primary harm type for the individual changed from one substantiation to the next). The conservative nature of this measure of multi-type maltreatment was acknowledged as a limitation in the study. This also resulted in a variation to the variables of focus within the replication. We carefully noted this variation to the methodology in our paper. Fortunately, this variation to the replication ensured that the study had increased relevance to the current research and policy environment.

Fourth, cross-sectional data taken from publicly available annual reports by the Queensland child protection department indicated changing rates in notifications and substantiations across Queensland over the time-frame of interest (i.e., from 1983 to 2008, or birth to age 18 years for each cohort). Additionally, the rates of notifications and substantiations of each maltreatment subtype had also changed over this period. We could only hypothesise that these changes to substantiations of each maltreatment subtype were partly attributable to changing knowledge and understanding of maltreatment subtypes and assessments of their potential impact on children, and possibly changing societal norms. We had no data with which we could test these hypotheses. Regardless, these cross-sectional figures indicated that we needed to exercise caution in the interpretation of results that related to the timing of maltreatment. For example, if notifications and substantiations increased over the life-course of a cohort, the longitudinal data could indicate higher rates of notifications in adolescence, which could affect observed links between maltreatment and offending. 

Additionally, these changing rates of substantiations across the life-course could be attributable to individual level factors or system factors. When interpreting our results, we carefully compared each cohort and considered potential age, period, and cohort effects. Fortunately, our examinations indicated that the changes were more evident in cross-sectional data than longitudinal data. This process of contextualising and evaluating the datasets confirmed that the results of the replication study were not artefact, and provided confirmation of the utility of replications using longitudinal administrative data for both theory and policy relevant child maltreatment research.

Fifth, in the earlier study we had access to both formal police cautions and appearances in youth court. In the replication study we only had access to the youth court appearance data, as the formal police cautioning data were not included in our data agreements at the time of data linkage. As diversion of young offenders is a priority in the Queensland youth justice system, we clearly acknowledged that offending as represented by court finalisations for the 1990 cohort provided a conservative estimate of offending, and likely represented more serious or persistent offenders. Importantly, the comparison of results across the 1983/1984 cohorts and the 1990 cohort appropriately focussed on general patterns rather than exact rates of offending across trajectory groups.

Despite the above listed challenges to our replication, our replication was extremely valuable. We confirmed the generalisability of the results obtained from longitudinal administrative data. In particular, our results showed that despite a changing child protection environment, relationships between maltreatment and offending within the jurisdiction remained stable over time. Hence, the process of replication bolstered confidence both in the utility of the data source, as well as the results themselves. Careful examination of each dataset to account for variations, consideration of the different contexts in which the data for each dataset were collected, acknowledgment of challenges relating to the replication method and the datasets, careful documentation of how key variables were created, and a focus on patterns rather than exact figures, were adequate techniques to address the challenges to replication using longitudinal linked administrative data. We are now in the process of extending our data linkage to incorporate data from additional government departments in Queensland [11], in the hopes of noting multiple system contacts of maltreated individuals, and links between maltreatment and various developmental outcomes including mental illness, adult offending, and domestic violence. Replication will remain a key element in our work. 

## 8. Conclusions

There is a clear need for longitudinal replication studies in the child maltreatment literature. Administrative data can be used to produce these longitudinal replication studies. With ever increasing power of computers, the quality and accessibility of administrative data are improving along with the available analytical techniques. However, it is important to acknowledge variations in data sources, the context from which each data source is drawn, and the ways in which these variations and contextual factors may impact on research results, conclusions, and policy suggestions. To assist future replication efforts, current researchers can carefully document their data restructuring and variable creation processes.

Despite the challenges associated with the use of administrative data for longitudinal replication studies of child maltreatment, it is important to acknowledge the numerous benefits. These data allow complex multilevel analyses [2], facilitate assessments of the generalisability of research results, are generally time and cost-effective [1,2], remove disclosure burden from victims [1], and can bridge the divide between research, policy, and practice [2]. Administrative data are already contributing to our knowledge base about child maltreatment. Continued responsible use of these data can contribute to greater knowledge, improvement of child protection policy and practice, and better outcomes for vulnerable children and their families.
